# A Class of Coning Algorithms Based on a Half-Compressed Structure

**DOI:** 10.3390/s140814289

**Published:** 2014-08-06

**Authors:** Chuanye Tang, Xiyuan Chen

**Affiliations:** Key Laboratory of Micro-Inertial Instrument and Advanced Navigation Technology, Ministry of Education, School of Instrument Science and Engineering, Southeast University, Sipailou 2, Nanjing 210096, China; E-Mail: 230129460@seu.edu.cn

**Keywords:** compressed algorithm, half-compressed algorithm, uncompressed algorithm, coning environment, maneuver environment

## Abstract

Aiming to advance the coning algorithm performance of strapdown inertial navigation systems, a new half-compressed coning correction structure is presented. The half-compressed algorithm structure is analytically proven to be equivalent to the traditional compressed structure under coning environments. The half-compressed algorithm coefficients allow direct configuration from traditional compressed algorithm coefficients. A type of algorithm error model is defined for coning algorithm performance evaluation under maneuver environment conditions. Like previous uncompressed algorithms, the half-compressed algorithm has improved maneuver accuracy and retained coning accuracy compared with its corresponding compressed algorithm. Compared with prior uncompressed algorithms, the formula for the new algorithm coefficients is simpler.

## 1. Introduction

In the recent decades since Jordan [1] and Bortz [2] introduced the two-stage attitude updating algorithm for strapdown inertial navigation systems (SINS), the design of efficient coning algorithms which include designing an efficient coning correction structure and achieving the optimized structure coefficients for coning correction accounting for a portion of the rotation vector has always been an attractive topic.

Jordan [1] first presented the two-sample algorithm structure for non-commutativity error compensation. Miller [3] first presented the three-sample algorithm structure and the concept of designing the coning correction algorithm for optimum performance in a pure coning environment by using a truncated coning frequency Taylor-series expansion formulation for updating the rotation vector errors corresponding to updating the quaternion error. On the basis of Miller's idea, Ignagni [4] summarized the generally uncompressed algorithm structure for coning correction and proposed several coning correction algorithms. Lee [5] applied Miller's idea and concluded that there exists redundancy in the uncompressed coning correction structure under coning motion conditions. Based on Lee's conclusion, Ignagni [6] proved that the cross product of both integral angular rate samples is independent of absolute time and a function of merely the relative time interval between sampling points under coning motion conditions, and derived the first compressed coning correction structure. Different from the previous coning algorithms for gyro error-free outputs, Mark [7] disclosed a method of tuning high-order coning algorithms to match the frequency response characteristics of gyros with filtered outputs. Based on the compressed coning correction structure, Savage [8] further expanded Miller's idea, and raised an idea of using the least square method to design the coning correction algorithm for balanced coning performance in a given discrete coning environment. Song [9] concentrated on the improvement of maneuver accuracy of coning algorithms, and developed an approach for recovering maneuver accuracy in previous coning algorithms based on the uncompressed structure by combining the earliest time Taylor-series method and the latest frequency methods.

This paper proposes a new half-compressed coning correction structure which is analytically proven to be equivalent to the traditional compressed coning correction structure under coning motion conditions. On the basis of the equivalency of the two types of structures, the half-compressed algorithm coefficients can be derived directly from the past compressed coning algorithm coefficients, rather than being specifically designed for coning or maneuver environments. The building of a reasonable structure makes for a simpler formula for the coefficients, retained coning accuracy and improved maneuver performance for the new half-compressed algorithm.

## 2. Attitude Algorithm Structure

The classical attitude updating computation formula [1,2,8] in modern strapdown inertial navigation systems is given by:

$$\begin{array}{c} C_{b(l)}^{n}=C_{b(l-1)}^{n}\cdot C_{b(l)}^{b(l-1)}\\ C_{b(l)}^{b(l-1)}=I+f_{1}(\phi_{l})(\phi_{l}\times )+f_{2}(\phi_{l}){\left(\phi_{l}\times \right)}^{2}\\ f_{1}(\phi_{l})=\frac{\sin\left|\phi_{l}\right|}{\left|\phi_{l}\right|}=\sum_{i=1}{\left(-1\right)}^{i-1}\frac{{\left|\phi_{l}\right|}^{2(i-1)}}{2(i-1)!}\\ f_{2}(\phi_{l})=\frac{1-\cos\left|\phi_{l}\right|}{{\left|\phi_{l}\right|}^{2}}=\sum_{i=1}{\left(-1\right)}^{i-1}\frac{{\left|\phi_{l}\right|}^{2(i-1)}}{2(i)!} \end{array}$$

(1) $$\phi_{l}\times =\left[\begin{array}{ccc} 0 & -\phi_{z} & \phi_{y}\\ \phi_{z} & 0 & -\phi_{x}\\ -\phi_{y} & \phi_{x} & 0 \end{array}\right]$$

where *n* is a navigation coordinate frame, *b* is a body coordinate frame, $C_{b(l)}^{n}$ and $C_{b(l-1)}^{n}$ are respectively an attitude direction cosine matrix at the end of attitude updating cycle *l* and cycle *l*−1, $C_{b(l)}^{b(l-1)}$ and *ϕ_l_* used to update $C_{b(l)}^{n}$ from $C_{b(l-1)}^{n}$ are respectively an updating attitude direction cosine matrix and an updating rotation vector from the ending time of cycle *l*−1 to the ending time of cycle *l*, |*ϕ_l_*| is the magnitude of vector *ϕ_l_*, and *ϕ_l_*× is the cross-product antisymmetry matrix composed of *ϕ_l_* components. The rotation vector *ϕ_l_* for the attitude update is generally calculated by using a simple form to approximate the integral of the rotation vector differential equation. A commonly used single-speed form [1,4,6,7] is given by:

(2) $$\begin{array}{c} \phi_{l}=\alpha_{l}+\delta \phi_{l},\\ \alpha_{l}=\alpha (t_{l},t_{l-1}),\\ \delta \phi_{l}=\frac{1}{2}\int_{t_{l-1}}^{t_{l}}\alpha (t,t_{l-1})\times \omega dt,\\ \alpha (t,t_{l-1})=\int_{t_{l-1}}^{t}\omega dt \end{array}$$

where *t* is a time, *α_l_* is the integral of the gyro sensed angular rate *ω* from time *t_l_*_−1_ to time *t_l_*, and *δϕ_l_* denotes the coning correction.

## 3. Coning Correction Structure

In recent decades, strapdown attitude algorithm design activity has centered on developing routines for computing the coning correction using various approximations to the updating rotation vector *ϕ_l_* in Equation (2). The traditional numerical computation algorithm formula for updating rotation vector *ϕ_l_* has the form of the integrated angular rate *α_l_* and the coning correction $\delta {\hat{\phi }}_{l}$ [4,6,8,9]:

(3) $$\begin{array}{c} \phi_{l}=\alpha_{l}+\delta {\hat{\phi }}_{l},\\ \alpha_{l}=\sum_{k=N-N+1}^{N}\Delta \alpha_{k},\\ \delta {\hat{\phi }}_{l}=\sum_{i=1}^{N-1}\sum_{j=i+1}^{N}\varsigma_{ij}\Delta \alpha_{i}\times \Delta \alpha_{j} \end{array}$$

where each Δ*α* is an angular increment sample over a fixed time interval *T_k_*, the Δ*α* s are adjacent and spaced sequentially forward in time, Δ*α_N_*_−_*_L_*_+_*_1_* begins at time *t_l_*_−1_, Δ*α_N_* ends at time *t_l_*, *ς_ij_* s are coefficients depending on the coning correction form, *L* is the number of angular increment samples selected to compute *α_l_* in cycle *l*, *N* selected to be equal to or greater than *L* is the number of angular increment samples selected to compute $\delta {\hat{\phi }}_{l}$ in cycle *l*. This form is the well-known uncompressed *N* subsample algorithm form with angular increments.

Based on the pure coning motion properties, the compressed algorithm form [6,8] equivalent to the uncompressed form for the coning correction $\delta {\hat{\phi }}_{l}$ in Equation (3) is given by:

(4) $$\delta {\hat{\phi }}_{l}=\sum_{s=1}^{N-1}K_{s}\Delta \alpha_{N-s}\times \Delta \alpha_{N},K_{s}=\sum_{i=s+1}^{N}\varsigma_{i-s,i}$$

where *K_s_* is the coning correction coefficient equivalent to the sum of *ς_i_*_−_*_s,i_* s from Equation (3), and other signs are defined as those in Equation (3).

**Figure 1.**
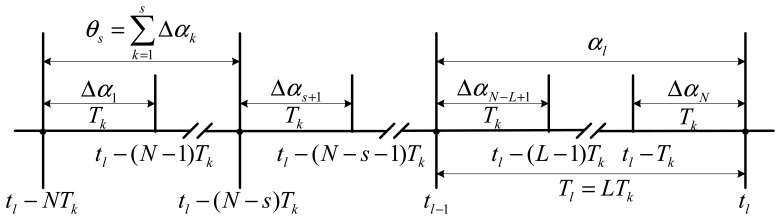
Distribution of several kinds of angular increment series against time.

Both traditional coning correction forms defined by Equations (3) and (4) are equivalent under coning motion conditions, but not equivalent under maneuver conditions. Song [9] indicated that the algorithms based on the uncompressed form of Equation (3) would give much higher maneuver accuracies, but have a much heavier computation load than those based on the compressed form of Equation (4) after intensive design under maneuver conditions. This paper proposes a half-compressed algorithm form different from the former forms for coning correction $\delta {\hat{\phi }}_{l}$ given by:

(5) $$\delta {\hat{\phi }}_{l}=\sum_{s=1}^{N-1}J_{s}\theta_{s}\times \Delta \alpha_{s+1},\theta_{s}=\sum_{k=1}^{s}\Delta \alpha_{k}$$

where *J_s_* is the coning correction coefficient depending on the half-compressed structure, *θ_s_* which can be directly achieved from the process of computing *α_l_* in Equation (3) is an angular increment beginning at time *t_l_*−*NT_k_* and ending at time *t_l_*−(*N*−*s)T_k_*, *T_k_* is the angular increment sample time interval, and other signs are defined as those in Equation (3). Several kinds of angular increments and time intervals defined by Equations (3)–(5) are illustrated in Figure 1.

## 4. Coning Correction Structure Equivalency and Algorithm Design

Assume that the body is undergoing the pure coning motion defined by the angular rate vector [4,6,8]:

(6) ω(t)=aΩcos(Ωt)I+bΩsin(Ωt)J

where *t* is a time, *ω*(*t*) is an angular rate vector in the body frame at time *t*, *α* and *b* are amplitudes of the angular oscillations in two orthogonal axes of the body, Ω is frequency associated with the angular oscillations, and ***I***, ***J*** are unit vectors along the two orthogonal axes of the body.

Under the coning motion defined by Equation (6), Ignagni [4] had derived the cross product Δ*α_i_* × Δ*α_j_*:

(7) $$\begin{array}{c} \Delta \alpha_{i}\times \Delta \alpha_{j}=abf_{j-i}(\beta )\text{K}\\ f_{j-i}(\beta )\equiv 2\sin[(j-i)\beta ]-\sin[(j-i-1)\beta ]-\sin[(j-i+1)\beta ],\\ \beta \equiv \Omega T_{k} \end{array}$$

where ***K*** is an unit vector orthogonal to the unit vectors ***I*** and ***J***, and *β* is a coning frequency parameter relevant to *T_k_*.

Simplification of the coning algorithm form of Equation (3) in the form of Equation (4) in [6], utilizing the coning property expressed by Equation (7) also allows the coning algorithm form of Equation (5) to be simplified as the form of Equation (4) with the relationship of coefficients *J_s_* s and *K_s_* s:

(8) $$\begin{array}{c} C_{N-1}\cdot A=B,\\ A={\left(a_{s,1}\right)}_{(N-1)\times 1},a_{s,1}\equiv J_{s},\\ B={\left(b_{s,1}\right)}_{(N-1)\times 1},b_{s,1}\equiv K_{s},\\ C_{1}=1,C_{2}=\left(\begin{array}{ll} 1 & 1\\ & 1 \end{array}\right),C_{3}=\left(\begin{array}{lll} 1 & 1 & 1\\ & 1 & 1\\ & & 1 \end{array}\right),\\ C_{N-1}={\left(\begin{array}{llll} 1 & 1 & \cdots & 1\\ & 1 & \ddots & \vdots \\ & & \ddots & 1\\ & & & 1 \end{array}\right)}_{\left(N-1)\times (N-1\right)},N\geq 5 \end{array}$$

where *A* is *N*−1 by one column matrix formed from components *J_s_* s, *B* is *N*−1 by one column matrix formed from components *K_s_*s, and *C_N_*_−1_ is *N*−1 by *N*−1 matrix whose upper triangular components are ones, and others are zeros concealed in Equation (8).

It is easily proved that *C_N_*_−1_ is a non-singular matrix. Thus *A* and *B* are linear representation. That means, N-sample coning algorithms designed by using the same optimum method and based on the correction structures of Equation (4) and Equation (5) have the same coning correction value under pure a coning motion. Therefore, Equation (5) and Equation (4) are equivalent under coning motions. Solving Equation (8) results in:

(9) $$A={\left(C_{N-1}\right)}^{-1}B$$

which gives the optimized coefficients *J_s_* s applicable to the form of Equation (5) from already designed *K_s_* s of Equation (4).

## 5. Algorithm Performance Evaluation

Above we have verified that the half-compressed structure of Equation (5) and the compressed structure of Equation (4) are equivalent under pure coning motion. The uncompressed algorithm based on Equation (3) presented by Song [9] also has the same accuracy as the compressed algorithm based on Equation (4) under a coning motion. Thus, the algorithms designed by using the same optimum method and based on Equations (3)–Equation (5) have the same coning correction accuracy under a pure coning environment.

Below is an error model used for evaluating the coning algorithm accuracy under maneuver environments. Assume that the body is undergoing a maneuver angular motion characterized by the angular rate vector:

(10) $$\omega (t)=\sum_{j=1}^{M}{\bar{g}}_{j}t^{j-1}$$

where ${\bar{g}}_{j}$ is a coefficient vector based on the form of Equation (10) *M* is the coefficient vector number, *t* is a time. To evaluate the algorithm accuracy in maneuver environments, Equation (10) can be rewritten as another equivalent form:

(11) $$\omega (t)=\sum_{i=1}^{M}g_{i}{\left(t-t_{l-1}\right)}^{i-1}$$

where *g_j_* is a coefficient vector based on the form of Equation (11).

Through investigating Equation (10) and Equation (11), we can get the relationship of *g_j_* s and *ḡ_j_* s:

(12) $$\begin{array}{c} G\Gamma (t_{l-1})=\bar{G},G\equiv {\left(g_{i}\right)}_{M\times 1},\bar{G}\equiv {\left({\bar{g}}_{j}\right)}_{M\times 1},\Gamma (t_{l-1})\equiv {\left(\gamma_{ji}(t_{l-1})\right)}_{M\times M}\\ \gamma_{ji}(t_{l-1})=\{\begin{array}{cc} {\left(-t_{l-1}\right)}^{i-j}, & j=1\\ {\left(-t_{l-1}\right)}^{i-j}(i-1)!/(j-1)!, & 1<j\leq i\\ 0, & j>i \end{array} \end{array}$$

where Γ (*t_l_*_−1_) is a *M* by *M* square matrix whose the *j* th row and *i* th column component is *γ_ji_* (*t_l_*_−1_), *G* is a *M* by one column matrix whose the *i* th row component is *g_i_*, and $\bar{G}$ is a *M* by one column matrix whose the *j* th row component is ${\bar{g}}_{j}$.

According to the maneuver error analysis method given by [9], the error of a coning algorithm based on uncompressed correction structure of Equation (3) under the maneuver motion expressed by Equation (11) with *M* ≥ 5 can be built as:

(13) $$\begin{array}{c} e_{m}(t)=\delta {\hat{\phi }}_{l}-\delta \phi_{l}=z_{3}g_{1}\times g_{2}{\left(t-t_{l-1}\right)}^{3}+z_{4}g_{1}\times g_{3}{\left(t-t_{l-1}\right)}^{4}\\ +(z_{51}g_{1}\times g_{4}+z_{52}g_{2}\times g_{3}){\left(t-t_{l-1}\right)}^{5}+(z_{61}g_{1}\times g_{5}+z_{62}g_{2}\times g_{4}){\left(t-t_{l-1}\right)}^{6},\\ +(z_{71}g_{1}\times g_{6}+z_{72}g_{2}\times g_{5}+z_{73}g_{3}\times g_{4}){\left(t-t_{l-1}\right)}^{7}+o\left({\left(t-t_{l-1}\right)}^{8}\right)\\ z_{3}=\frac{1}{6}\left(f_{3}-\frac{1}{2}\right),z_{4}=\frac{1}{12}(f_{4}-1),z_{51}=\frac{1}{20}\left(f_{51}-\frac{3}{2}\right),z_{52}=\frac{1}{60}(f_{52}-1),z_{61}=\frac{1}{30}(f_{61}-2),\\ z_{62}=\frac{1}{120}\left(f_{62}-\frac{5}{2}\right),z_{71}=\frac{1}{42}\left(f_{71}-\frac{5}{2}\right),z_{72}=\frac{1}{210}\left(f_{72}-\frac{9}{2}\right),z_{73}=\frac{1}{420}\left(f_{73}-\frac{5}{2}\right) \end{array}$$

where:

(14) $$\begin{array}{c} f_{3}=\frac{6}{L^{3}}\sum_{i=1}^{N-1}\sum_{j=i+1}^{N}\varsigma_{ij}(j-i)\\ f_{4}=\frac{12}{L^{4}}\sum_{i=1}^{N-1}\sum_{j=i+1}^{N}\varsigma_{ij}(j-i)(2L-2N+i+j-1)\\ f_{51}=\frac{5}{L^{5}}\sum_{i=1}^{N-1}\sum_{j=i+1}^{N}\varsigma_{ij}[{\left(L-N+j\right)}^{4}-{\left(L-N+j-1\right)}^{4}-{\left(L-N+j\right)}^{4}+{\left(L-N+j-1\right)}^{4}]\\ f_{52}=\frac{10}{L^{5}}\sum_{i=1}^{N-1}\sum_{j=i+1}^{N}\varsigma_{ij}(j-i)\left[3(2L-2N+i+j-2)+1+6\left(L-N+i-1\right)\left(L-N+j-1\right)\right]\\ f_{61}=\frac{6}{L^{6}}\sum_{i=1}^{N-1}\sum_{j=i+1}^{N}\varsigma_{ij}\left[{\left(L-N+j\right)}^{5}-{\left(L-N+j-1\right)}^{5}-{\left(L-N+i\right)}^{5}+{\left(L-N+i-1\right)}^{5}\right]\\ f_{62}=\frac{15}{L^{6}}\sum_{i=1}^{N-1}\sum_{j=i+1}^{N}\varsigma_{ij}\left[2(j-i)(2L-2N+i+j-1)\left[4(L-N+i-1)(L-N+j-1)+2(2L-2N+i+j-2)+1\right]\right]\\ f_{71}=\frac{7}{L^{7}}\sum_{i=1}^{N-1}\sum_{j=i+1}^{N}\varsigma_{ij}\left[{\left(L-N+j\right)}^{6}-{\left(L-N+j-1\right)}^{6}-{\left(L-N+i\right)}^{6}+{\left(L-N+i-1\right)}^{6}\right]\\ f_{72}=\frac{21}{L^{7}}\sum_{i=1}^{N-1}\sum_{j=i+1}^{N}\varsigma_{ij}\left\{\left[{\left(L-N+i\right)}^{2}-{\left(L-N+i-1\right)}^{2}\right]\left[{\left(L-N+j\right)}^{5}-{\left(L-N+j-1\right)}^{5}\right]-\left[{\left(L-N+i\right)}^{5}-{\left(L-N+i-1\right)}^{5}\right]\left[{\left(L-N+j\right)}^{2}-{\left(L-N+j-1\right)}^{2}\right]\right\}\\ f_{73}=\frac{35}{L^{7}}\sum_{i=1}^{N-1}\sum_{j=i+1}^{N}\varsigma_{ij}\left\{\left[{\left(L-N+i\right)}^{3}-{\left(L-N+i-1\right)}^{3}\right]\left[{\left(L-N+j\right)}^{4}-{\left(L-N+j-1\right)}^{4}\right]-\left[{\left(L-N+i\right)}^{4}-{\left(L-N+i-1\right)}^{4}\right]\left[{\left(L-N+j\right)}^{3}-{\left(L-N+j-1\right)}^{3}\right]\right\} \end{array}$$

According to Equations (3)–(5), Equation (13) can be used for analyzing the maneuver errors of coning algorithms based on the compressed correction structure of Equation (4) and the half-compressed correction structure of Equation (5), when the coefficients *K* s in Equation (4) and the coefficients *J* s in Equation (5) are respectively expanded into the coefficients *ς* s in Equation (3) with the following relationships:

(15) $$\varsigma_{r-s,r}=\{\begin{array}{cc} K_{s}, & s=1,2,\ldots ,r-1,r=N\\ 0, & s=1,2,\ldots ,r-1,r=1,2,\ldots ,N-1 \end{array}$$

(16) $$\varsigma_{r,s+1}=J_{s,\;}s=1,2,\ldots ,N-1,r=1,2,\ldots ,s$$

## 6. Algorithm Examples and Simulation

To illustrate the properties of coning algorithms, algorithm errors computed using the optimized coning correction coefficients designed by using the frequency Taylor-series method and least minimum square method would be produced, compared, and analyzed under coning environments and maneuver environments, each with *T_k_* = 0.001s, *T_l_* = *LT_k_* and *L* = *N*:

(1) FTSc indicates the coning algorithm based on the compressed form of Equation (4) taking the coefficients designed by using frequency Taylor-series method.
(2) LMSc indicates the coning algorithm based on the compressed form of Equation (4) taking the coefficients designed by using least minimum square method.
(3) FTShc indicates the coning algorithm based on the half-compressed form of Equation (5) taking the coefficients designed by using frequency Taylor-series method.
(4) LMShc indicates the coning algorithm based on the half-compressed form of Equation (5) taking the coefficients designed by using least minimum square method.
(5) FTSuc indicates the coning algorithm based on the uncompressed form of Equation (3) taking the coefficients designed by Song [9] using frequency Taylor-series method.
(6) LMSuc indicates the coning algorithm based on the uncompressed form of Equation (3) taking the coefficients designed by Song [9] using least minimum square method.
(7) X-N indicates the N-sample algorithm X (X respectively denote FTSc, LMSc, FTShc, LMShc, FTSuc and LMSuc).

Tables 1 and 2 respectively give the 3-to-5-sample FTSc and LMSc algorithm coefficients *K_s_* s [5,6,8,9]. Using Equation (9), we can obtain the 3-to-5-sample FTShc and LMShc algorithm coefficients *J_s_* s given in Tables 3 and 4 from coefficients *K_s_* s in Tables 1 and 2. According to Equation (15), expanding the coefficients *K_s_* s in Tables 1 and 2 give the expanded coefficients *ς* s in Tables 5 and 6 for maneuver accuracy evaluation. According to Equation (16), expanding the coefficients *J_s_* s in Tables 3 and 4 give the expanded coefficients *ς* s in Tables 7 and 8 for maneuver accuracy evaluation.

Tables 9 and 10 give the 3-to-5-sample FTSuc and LMSuc algorithm coefficients *ς* s designed by Song [9] from the coefficients *K_s_* s in Tables 1 and 2, respectively.

The coefficients *z*_3_, *z*_4_, *z*_51_, *z*_52_, *z*_61_, *z*_62_, *z*_71_, *z*_72_ and *z*_73_ depending on the power series terms in Equation (13) are calculated with the coefficients in Tables 5, 6, 7, 8, 9 and 10, and respectively listed in Tables 11, 12, 13, 14, 15 and 16.

**Figure 2.**
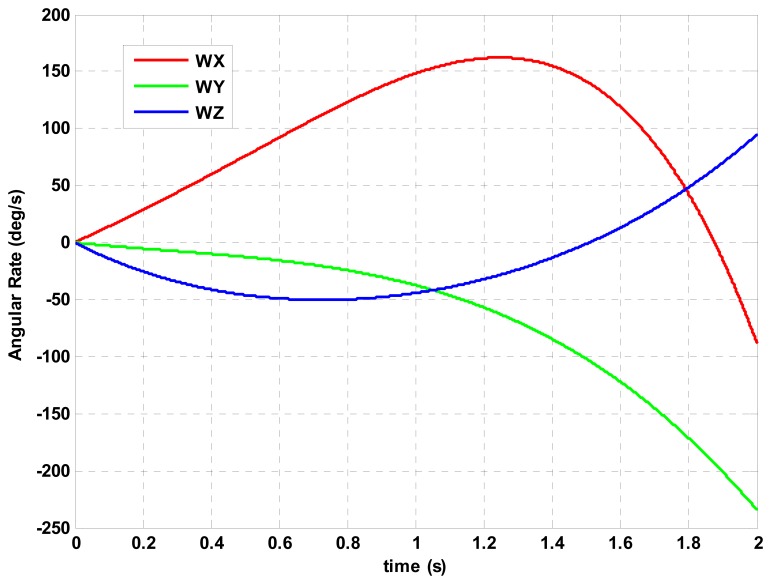
Maneuver angular rate (deg/s) *vs.* time (s).

The maneuver environment set for algorithm accuracy evaluation is the extreme 2 s angular rate profile pictured in Figure 2 with *M* = 5, ${\bar{g}}_{1}$ = [0 0 0]*^T^*, ${\bar{g}}_{2}$ = [19572/143 −4360/143 −21800/143]*^T^*, ${\bar{g}}_{3}$ = [1007/41 4000/143 9369/67]*^T^*, ${\bar{g}}_{4}$ = [4843/155 −4000/117 −9206/213]*^T^* and ${\bar{g}}_{5}$ = [−5813/131 − 625/858 3258/281]*^T^* in Equation (10). The dimension is deg/s for $\bar{g}$ s. According to Equation (13), the maneuver error vector *e_m_* (*t*) is computed for the compressed algorithms, the half-compressed algorithms and the uncompressed algorithms with Tables 5, 6, 7, 8, 9 and 10 coefficients over the Figure 2 maneuver profile.

Accordingly, maximum maneuver errors of several concerned algorithms over 2 s maneuver are listed in Table 17.

Comparing the data in Tables 11 to 16, it is indicated that the absolute values of *z*_3_ for FTSc, FTShc and FTSuc algorithms are zeros, LMSc, LMShc and LMSuc algorithms have the same non-zero *z*_3_, the absolute values of *z*_4_ for 3 to 5 sample FTShc (or LMShc) algorithms are respectively about one third, one sixth and one tenth those of *z*_4_ for 3 to 5 sample FTSc (or LMSc) algorithms, while the absolute values of *z*_4_ for 3 to 5 sample FTSuc (or LMSuc) algorithms are much smaller than those for 3 to 5 sample FTShc (or LMShc) algorithms. If the low order term with *z* in Equation (13) is the main supply of maneuver error, it would be concluded that the maneuver accuracy of FTShc algorithm is higher than that of FTSc algorithm if the FTSuc algorithm is compared with the FTShc algorithm, and the maneuver accuracy of LMShc algorithm is higher than that of LMSc algorithm if the LMSuc algorithm is compared with LMShc algorithm ignoring the error term with *z*_3_. The simulation results in Table 17 are basically consistent with the analytical conclusion above, whereas the maximum maneuver error of the LMSuc3 algorithm is bigger than that of the LMSc3 and LMShc3 algorithms owing to the coupling of the first two error terms with *z*_3_ and *z*_4_ under this particular 2 s maneuver condition.

According to the 21.3 μrad error contribution from sensors in Table 2 of [8] during the similar maneuver environment, all concerned algorithms with the maximum errors in Table 17 contributing less than 1% of 10 to 20 μrad are compatible with the overall INS accuracy requirement of 0.01 deg/h. Like the uncompressed algorithm (reference [9]), the half-compressed algorithm has significantly more accuracy than the past compressed algorithm, and the new algorithm is more than adequate for modern INS applications. The formula for the algorithm coefficients for the new half-compressed algorithm is simpler compared to the uncompressed algorithm.

## 7. Conclusions

The new half-compressed coning algorithm can be directly derived from the past compressed coning algorithm. The new algorithms are highly efficient overall in coning and maneuver environments. Compared with the past uncompressed algorithm, the formula for the new algorithm coefficients is simpler. Like the past uncompressed algorithm, the half-compressed algorithm and its corresponding compressed algorithm have the same coning accuracy, while the maneuver accuracy of the half-compressed algorithm is significantly higher than the past compressed algorithm, and more than adequate for modern INS applications.

## Figures and Tables

**Table 1.** FTSc algorithm coefficients.

*L*	*N*	*K*_1_	*K*_2_	*K*_3_	*K*_4_
3	3	27/20	9/20		
4	4	214/105	92/105	54/105	
5	5	1375/504	650/504	525/504	250/504

**Table 2.** LMSc algorithm coefficients.

*L*	*N*	*K*_1_	*K*_2_	*K*_3_	*K*_4_
3	3	1.360758	0.444312		
4	4	2.049323	0.866920	0.516734	
5	5	2.739618	1.277985	1.046872	0.495116

**Table 3.** FTShc algorithm coefficients.

*L*	*N*	*J*_1_	*J*_2_	*J*_3_	*J*_4_
3	3	18/20	9/20		
4	4	122/105	38/105	54/105	
5	5	725/504	125/504	275/504	250/504

**Table 4.** LMShc algorithm coefficients.

*L*	*N*	*J*_1_	*J*_2_	*J*_3_	*J*_4_
3	3	0.916446	0.444312		
4	4	1.182403	0.350186	0.516734	
5	5	1.461633	0.231113	0.551756	0.495116

**Table 5.** Coefficients expanded from the FTSc coefficients in Table 1.

*L*	*N*	**Coefficients**
3	3	*ς*_12_ = 0, *ς*_13_ = 9/12, *ς*_23_ = 27/20
4	4	ς_12_ = ς_13_ = ς_23_ = 0, ς_14_ = 54/105, ς_24_ = 92/105, ς_34_ = 214/105
5	5	*ς*_12_ = *ς*_13_ = *ς*_14_ = *ς*_23_ = *ς*_23_ = 0, *ς*_15_ = 250/504, *ς*_25_ = 525/504, *ς*_35_ = 650/504, *ς*_45_ = 1375/504

**Table 6.** Coefficients expanded from the LMSc coefficients in Table 2.

*L*	*N*	**Coefficients**
3	3	*ς*_12_ = 0, *ς*_13_ = 0.444312, *ς*_23_ = 1.360758
4	4	*ς*_12_ = *ς*_13_ = *ς*_23_ = 0, *ς*_14_ = 0.516734, *ς*_24_ = 0.866920, *ς*_34_ = 2.049323
5	5	*ς*_12_ = *ς*_13_ = *ς*_14_ = *ς*_23_ = *ς*_24_ = *ς*_34_ = 0, *ς*_15_ = 0.495116, *ς*_25_ = 1.046872, *ς*_35_ = 1.277985, *ς*_45_ = 2.739618

**Table 7.** Coefficients expanded from the FTShc coefficients in Table 3.

*L*	*N*	**Coefficients**
3	3	*ς*_12_ = 18/20, *ς*_13_ = *ς*_23_ = 9/20
4	4	*ς*_12_ = 122/105, *ς*_13_ = *ς*_23_ = 38/105, *ς*_14_ = *ς*_24_ = *ς*_34_ = 54/105
5	5	*ς*_12_ = 725/504, *ς*_13_ = *ς*_23_ = 125/504, *ς*_14_ = *ς*_24_ = *ς*_34_ = 275/504, *ς*_15_ = *ς*_25_ = *ς*_35_ = *ς*_45_ = 250/504

**Table 8.** Coefficients expanded from the LMShc coefficients in Table 4.

*L*	*N*	**Coefficients**
3	3	*ς*_12_ = 0.916446, *ς*_13_ = *ς*_23_ = 0.444312
4	4	*ς*_12_ = 1.182403, *ς*_13_ = *ς*_23_ = 0.350186, *ς*_14_ = *ς*_24_ = *ς*_34_ = 0.516734
5	5	*ς*_12_ = 1.461633, *ς*_13_ = *ς*_23_ = 0.231113, *ς*_14_ = *ς*_24_ = *ς*_34_ = 0.551756, *ς*_15_ = *ς*_25_ = *ς*_35_ = *ς*_45_ = 0.495116

**Table 9.** FTSuc algorithm coefficients.

*L*	*N*	**Coefficients**
3	3	*ς*_12_ = *ς*_23_ = 27/40, *ς*_13_ = 9/20
4	4	*ς*_12_ = *ς*_34_ = 232/315, *ς*_23_ = 178/315, *ς*_13_ = *ς*_24_ = 46/105, *ς*_14_ = 54/105
5	5	*ς*_12_ = 18575/24192, *ς*_13_ = 2675/6048, *ς*_14_ = 11,225/24,192, *ς*_15_ = 125/252, *ς*_23_ = 2575/6048, *ς*_24_ = 425/672, *ς*_25_ = 139,75/24,192, *ς*_34_ = 1975/3024, *ς*_35_ = 325/1512, *ς*_45_ = 21,325/24,192

**Table 10.** LMSuc algorithm coefficients.

*L*	*N*	**Coefficients**
3	3	*ς*_12_ = 0.681306, *ς*_13_ = 0.444312, *ς*_23_ = 0.679452
4	4	*ς*_12_ = 0.739716, *ς*_13_ = 0.432467, *ς*_14_ = 516734, *ς*_23_ = 0.571812, *ς*_24_ = 0.4434453, *ς*_34_ = 0.737795
5	5	*ς*_12_ = 769,240, *ς*_13_ = 0.438591, *ς*_14_ = 0.467191, *ς*_15_ = 0.495116, *ς*_23_ = 0.431753, *ς*_24_ = 0.625867, *ς*_25_ = 0.579681, *ς*_34_ = 0.656805, *ς*_35_ = 0.213527, *ς*_45_ = 0.881820

**Table 11.** Main attribution to maneuver error *e_m_* (*t*) for FTSc algorithm.

*L*	*N*	*z*_3_	*z*_4_	*z*_51_	*z*_52_	*z*_61_	*z*_62_	*z*_71_	*z*_72_	*z*_73_
3	3	0	1/60	13/540	13/1620	7/270	5/432	257/10,206	150/12,179	47/13,124
4	4	0	51/2240	55/1536	55/4608	187/4481	394/20,567	801/18,391	147/6512	89/12,840
5	5	0	83/3150	167/3901	77/5396	137/2657	79/3334	486/8749	515/17,789	193/21,636

**Table 12.** Main attribution to maneuver error *e_m_* (*t*) for LMSc algorithm.

*L*	*N*	*z*_3_	*z*_4_	*z*_51_	*z*_52_	*z*_61_	*z*_62_	*z*_71_	*z*_72_	*z*_73_
3	3	−2.29e−5	1.68e−2	2.42e−2	8.13e−3	2.61e−2	1.17e−2	2.54e−2	1.25e−2	3.64e−3
4	4	4.95e−7	2.28e−2	3.59e−2	1.19e−2	4.18e−2	1.92e−2	4.36e−2	2.26e−2	6.95e−3
5	5	1.07e−8	2.64e−2	4.28e−2	1.43e−2	5.16e−2	2.37e−2	5.56e−2	2.90e−2	8.93e−3

**Table 13.** Main attribution to maneuver error *e_m_* (*t*) for FTShc algorithm.

*L*	*N*	*z*_3_	*z*_4_	*z*_51_	*z*_52_	*z*_61_	*z*_62_	*z*_71_	*z*_72_	*z*_73_
3	3	0	−1/180	−1/108	−1/324	−1/90	−11/2160	−121/10,206	−56/9029	−403/204,120
4	4	0	−13/3360	−1/192	−1/576	−41/7680	−115/44,239	−47/9216	−23/7680	−91/92,160
5	5	0	−17/6300	−1/300	−1/900	−44/13,125	−16/9683	−37/11,250	−73/37,500	−29/45,000

**Table 14.** Main attribution to maneuver error *e_m_* (*t*) for LMShc algorithm.

*L*	*N*	*z*_3_	*z*_4_	*z*_51_	*z*_52_	*z*_61_	*z*_62_	*z*_71_	*z*_72_	*z*_73_
3	3	−2.29e−5	−5.85e−3	−9.69e−3	−3.18e−3	−1.16e−2	−5.23e−3	−1.23e−2	−6.36e−3	−2.02e−3
4	4	4.95e−7	−3.90e−3	−5.20e−3	−1.73e−3	−5.28e−3	−2.58e−3	−5.00e−3	−2.96e−3	−9.78e−4
5	5	1.07e−8	−2.71e−3	−3.33e−3	−1.11e−3	−3.35e−3	−1.65e−3	−3.29e−3	−1.95e−3	−6.45e−4

**Table 15.** Main attribution to maneuver error *e_m_* (*t*) for FTSuc algorithm.

*L*	*N*	*z*_3_	*z*_4_	*z*_51_	*z*_52_	*z*_61_	*z*_62_	*z*_71_	*z*_72_	*z*_73_
3	3	0	0	−1/1080	−1/3240	−1/540	−1/1080	−53/20,412	−107/68,040	−17/29,038
4	4	0	0	0	0	0	0	−1/16,128	−1/13,440	−1/32,256
5	5	0	0	0	0	0	0	−11/315,000	0	3/859,091

**Table 16.** Main attribution to maneuver error *e_m_* (*t*) for LMSuc algorithm.

*L*	*N*	*z*_3_	*z*_4_	*z*_51_	*z*_52_	*z*_61_	*z*_62_	*z*_71_	*z*_72_	*z*_73_
3	3	−2.29e−5	0	−9.12e−4	−2.56e−4	−1.83e−3	−8.46e−4	−2.57e−3	−1.48e−3	−5.53e−4
4	4	4.95e−7	−1.30e−8	−2.00e−8	−1.04e−6	1.32e−7	−1.02e−8	−6.17e−5	−7.30e−5	−2.84e−5
5	5	1.07e−8	1.07e−9	2.24e−9	2.21e−8	3.03e−9	1.55e−9	−3.49e−5	2.08e−9	3.45e−6

**Table 17.** Maximum maneuver error over 2 s maneuver.

*L*	*N*	**Maximum Maneuver Error, *μ* rad**					

		**FTSc**	**LMSc**	**FTShc**	**LMShc**	**FTSuc**	**LMSuc**
3	3	1.00e−2	−1.88e−2	−3.34e−3	3.65e−3	2.86e−6	−2.52e−2
4	4	3.24e−2	3.25e−2	−5.51e−3	−5.54e−3	1.48e−12	9.66e−4
5	5	7.32e−2	7.33e−2	−7.50e−3	−7.52e−3	−7.23e−13	3.25e−5
