# Correction: Tang, C. Y. and Chen, X.Y. A Class of Coning Algorithms Based on a Half-Compressed Structure. 

**DOI:** 10.3390/s150204425

**Published:** 2015-02-13

**Authors:** Chuanye Tang, Xiyuan Chen

**Affiliations:** Key Laboratory of Micro-Inertial Instrument and Advanced Navigation Technology, Ministry of Education, School of Instrument Science and Engineering, Southeast University, Sipailou 2, Nanjing 210096, China; E-Mail: 230129460@seu.edu.cn

## 1. Change in Tables/Equations

Due to an oversight by MDPI and the authors, the following numerical corrections were not made in the originally published article [1]. MDPI-Sensors and the authors would like to apologize for any inconvenience brought to the readers.

The authors wish to make the following correction to the article [1]:

The former Table 9 (labelled here as Old Table 9) and Table 10 (labelled here as Old Table 10) should be replaced by the new versions shown below (labelled here as New Table 9 and New Table 10), respectively. The *z* s in Tables 15 and 16 and the maneuver errors in [1] Table 17 will not be affected by the correction to Tables 9 and 10, because these *z* s and the maneuver errors were all calculated using the correct coefficients in New Tables 9 and 10. That means, the mistakes in Old Tables 9 and 10 are just writing errors.

**Old Table 9.** FTSuc algorithm coefficients.

***L***	***N***	**Coefficients**
3	3	ς_12_ = ς_23_ = 27/40, ς_13_ = 9/20
4	4	ς_12_ = ς_34_ = 232/315, ς_23_ = 178/315, ς_13_ = ς_24_ = 46/105, ς_14_ = 54/105
5	5	ς_12_ = 18575/24192, ς_13_ = 2675/6048, ς_14_ = 11,225/24,192, ς_15_ = 125/252, ς_23_ = 2575/6048, ς_24_ = 425/672, ς_25_ = 139,75/24,192, ς_34_ = 1975/3024, ς_35_ = 325/1512, ς_45_ = 21,325/24,192

**New Table 9.** FTSuc algorithm coefficients.

***L***	***N***	**Coefficients**
3	3	ς_12_ = ς_23_ = 27/40, ς_13_ = 9/20
4	4	ς_12_ = ς_34_ = 232/315, ς_23_ = 178/315, ς_13_ = ς_24_ = 46/105, ς_14_ = 54/105
5	5	ς_12_ = 21325/24192, ς_13_ = 325/1512, ς_14_ = 13975/24192, ς_15_ = 125/252, ς_23_ = 1975/3024, ς_24_ = 425/672, ς_25_ =11225/24192, ς_34_ =2575/6048, ς_35_ =2675/6048, ς_45_ = 18575/24192

**Old Table 10.** LMSuc algorithm coefficients.

***L***	***N***	**Coefficients**
3	3	ς_12_ = 0.681306, ς_13_ = 0.444312, ς_23_ = 0.679452
4	4	ς_12_ = 0.739716, ς_13_ = 0.432467, ς_14_ = 516734, ς_23_ = 0.571812, ς_24_ = 0.4434453, ς_34_ = 0.737795
5	5	ς_12_ = 769,240, ς_13_ = 0.438591, ς_14_ = 0.467191, ς_15_ = 0.495116, ς_23_ = 0.431753, ς_24_ = 0.625867, ς_25_ = 0.579681, ς_34_ = 0.656805, ς_35_ = 0.213527, ς_45_ = 0.881820

**New Table 10.** LMSuc algorithm coefficients.

***L***	***N***	**Coefficients**
3	3	ς_12_ = 0.679452, ς_13_ = 0.444312, ς_23_ = 0.681306
4	4	ς_12_ = 0.737795, ς_13_ = 0.434453, ς_14_ = 516734, ς_23_ = 0.571812, ς_24_ =0.432467, ς_34_ = 0.739716
5	5	ς_12_ = 0.881820, ς_13_ = 0.213527, ς_14_ = 0.579681, ς_15_ = 0.495116, ς_23_ = 0.656805, ς_24_ = 0.625867, ς_25_ =0.467191, ς_34_ =0.431753, ς_35_ =0.438591, ς_45_ = 0.769240

The former Equation (12) of [1]:

(12) $$\begin{array}{c} G\Gamma (t_{l-1})=\bar{G},G\equiv {\left(g_{i}\right)}_{M\times 1},\bar{G}\equiv {\left({\bar{g}}_{j}\right)}_{M\times 1},\Gamma (t_{l-1})\equiv {\left(\gamma_{ji}(t_{l-1})\right)}_{M\times M}\\ \gamma_{ji}\left(t_{l-1}\right)=\{\begin{array}{ccc} {\left(-t_{l-1}\right)}^{i-j} & , & j=1\\ {\left(-t_{l-1}\right)}^{i-j}(i-1)!/(j-1)! & , & 1<j\leq i\\ 0 & , & j>i \end{array} \end{array}$$

Should be replaced by the new Equation (12):

(12) $$\begin{array}{c} G\Gamma (t_{l-1})=\bar{G},G\equiv {\left(g_{i}\right)}_{M\times 1},\bar{G}\equiv {\left({\bar{g}}_{j}\right)}_{M\times 1},\Gamma (t_{l-1})\equiv {\left(\gamma_{ji}(t_{l-1})\right)}_{M\times M}\\ \gamma_{ji}\left(t_{l-1}\right)=\{\begin{array}{ccc} {\left(-t_{l-1}\right)}^{i-j} & , & j=1\\ {\left(-t_{l-1}\right)}^{i-j}(i-1)!/\left((i-j)!(j-1)!\right) & , & 1<j\leq i\\ 0 & , & j>i \end{array} \end{array}$$

Affected by the correction to Equation (12), the former Table 17 (labelled here as Old Table 17) of [1] should be replaced by the new version (labelled here as New Table 17). The correction to Table 17 will not affect the conclusions of [1].

**Old Table 17.** Maximum maneuver error over 2 s maneuver.

***L***	***N***	**Maximum Maneuver Error, μ Rad**					

		**FTSc**	**LMSc**	**FTShc**	**LMShc**	**FTSuc**	**LMSuc**
3	3	1.00e–2	−1.88e–2	−3.34e–3	3.65e–3	2.86e–6	−2.52e–2
4	4	3.24e–2	3.25e–2	−5.51e–3	−5.54e–3	1.48e–12	9.66e–4
5	5	7.32e–2	7.33e–2	−7.50e–3	−7.52e–3	−7.23e–13	3.25e–5

**New Table 17.** Maximum maneuver error over 2 s maneuver.

***L***	***N***	**Maximum Maneuver Error, μ rad**					

		**FTSc**	**LMSc**	**FTShc**	**LMShc**	**FTSuc**	**LMSuc**
3	3	−1.09e–2	−7.29e–3	3.63e–3	7.48e–3	1.39e–6	3.66e–3
4	4	−3.52e–2	−3.54e–2	5.98e–3	5.89e–3	1.67e–12	−1.40e–4
5	5	−7.97e–2	−7.97e–2	8.15e–3	8.17e–3	−3.09e–13	−4.73e–6

## 2. Change in Main Body Paragraphs

Due to an obscurity on how Equations (13) and (14) of [1] were built, the authors wish to insert some additional sentences to explain how Equations (13) and (14) of [1] can be converted from Equations (59) and (13) of Song (reference [9] of [1]).

Below we respectively denote the Song ς*_ij_* and the [1] ς*_ij_* using (ς*_ij_*)*_S_* and (ς*_ij_*)*_T_*.

After setting *p* = *N* + 1− *i* and *q* = *N* + 1− *j*, we can rewrite Equation (5) of Song [9] as:

(a1) $$\delta {\hat{\phi }}_{\text{unc}}(t)=\sum_{p=1}^{N-1}\sum_{q=p+1}^{N}{\left(\varsigma_{N+1-p,N+1-q}\right)}_{S}\Delta \alpha_{p}\times \Delta \alpha_{q}$$

If δϕ̂*_unc_*(*t*) and (ς*_N_*_+1−_*_p,N_*_+1−_*_q_*)*_S_* are respectively denoted by δϕ̂*_l_* and ξ*_pq_*, Equation (a1) can be rewritten as:

(a1) $$\delta {\hat{\phi }}_{l}=\sum_{p=1}^{N-1}\sum_{q=p+1}^{N}\xi_{pq}\Delta \alpha_{p}\times \Delta \alpha_{q}$$

Comparing Equation (a2) with the [1] Equation (3), we will find that both equations are the same expression under ξ*_pq_* = (ς*_ij_*)*_T_* with *p* = *i* and *q* = *j*.

Thus, to make Song [9] Equation (5) of and [1] Equation (3) equivalent will achieve (ς*_ij_*)*_T_* = (ς*_N_*_+1−_*_i_*_,_*_N_*_+1−_*_j_*)*_S_*. Using this relationship, we have respectively converted ς*_ij_* s in Tables 1 and 2 of Song [9] to ς*_ij_* s in New Tables 9 and 10, also we can convert Song [9] Equation (13) to [1] Equation (14), when Song [9] *n* is replaced by *L*.

Now we rewrite Song [9] Equation (59) as:

(a3) $$\begin{array}{c} \begin{array}{l} \delta {\hat{\phi }}_{\text{unc}}-\delta \phi_{c}=z_{3}^{\prime }\omega (t_{m-1})\times \dot{\omega }(t_{m-1}){\left(t-t_{m-1}\right)}^{3}+z_{4}^{\prime }\omega (t_{m-1})\times \ddot{\omega }(t_{m-1}){\left(t-t_{m-1}\right)}^{4}\\ +\left(z_{51}^{\prime }\omega (t_{m-1})\times \dddot{\omega }(t_{m-1})+z_{52}^{\prime }\dot{\omega }(t_{m-1})\times \ddot{\omega }(t_{m-1})\right){\left(t-t_{m-1}\right)}^{5}\\ +\left(z_{61}^{\prime }\omega (t_{m-1})\times \dot{\dddot{\omega }}(t_{m-1})+z_{62}^{'}\dot{\omega }(t_{m-1})\times \dddot{\omega }(t_{m-1})\right){\left(t-t_{m-1}\right)}^{6}\\ +\left(z_{71}^{'}\omega (t_{m-1})\times \ddot{\dddot{\omega }}(t_{m-1})+z_{72}^{'}\dot{\omega }(t_{m-1})\times \dot{\dddot{\omega }}(t_{m-1})+z_{73}^{'}\ddot{\omega }(t_{m-1})\times \dddot{\omega }(t_{m-1})\right){\left(t-t_{m-1}\right)}^{7}\\ +o\left({\left(t-t_{m-1}\right)}^{9}\right) \end{array}\\ z_{3}^{\prime }=\frac{1}{6}\left(f_{3}-\frac{1}{2}\right),z_{4}^{\prime }=\frac{1}{24}(f_{4}-1),z_{51}^{\prime }=\frac{1}{120}\left(f_{51}-\frac{3}{2}\right),z_{52}^{\prime }=\frac{1}{120}(f_{52}-1),z_{61}^{\prime }=\frac{1}{720}(f_{61}-2)\\ z_{62}^{\prime }=\frac{1}{720}\left(f_{62}-\frac{5}{2}\right),z_{71}^{\prime }=\frac{1}{5040}\left(f_{71}-\frac{5}{2}\right),z_{72}^{\prime }=\frac{1}{5040}\left(f_{72}-\frac{9}{2}\right),z_{73}^{\prime }=\frac{1}{5040}\left(f_{73}-\frac{5}{2}\right) \end{array}$$

where *f* s are of Song [9], rather than of [1].

Set:

(a4) $$g_{i}=\frac{d^{i-1}}{dt^{i-1}}\left({\omega (t)|}_{t=t_{m-1}}\right)/(i-1)!,i=1,2,\ldots$$

where *i* is a positive integer, and $\frac{d^{0}}{dt^{0}}\left({\omega (t)|}_{t=t_{m-1}}\right)$ denotes ω(*t_m_*_−1_).

Then Equation (a3) can be converted into [1] Equation (13), when δϕ̂*_unc_*(*t*) − δϕ*_c_*(*t*), *t_m_*_−1_ and *n* are respectively replaced by δϕ̂*_l_* − δϕ*_l_*, *t_l_*_−1_ and *L*.

To confirm the correctness of [1] Equations (13) and (14), the *z* s in [1] Equation (13) are calculated for LMSuc using the [1] *f* s (see [1] Equation (14)) and ς s in New Table 10. Also the *z*^′^ s in Equation (a3) are calculated for UncExp using the Song [9] *f* s (see Song [9] Equations (13)) and ς s in Song [9] Table 1. The *z* s for LMSuc and the *z*^′^ s for UncExp are listed in Tables a1 (the copy of [1] Table 16) and a2, respectively.

**Table a1.** The *z* s for [1] LMSuc.

***L***	***N***	***z*_3_**	***z*_4_**	***z*_51_**	***z*_52_**	***z*_61_**	***z*_62_**	***z*_71_**	***z*_72_**	***z*_73_**
3	3	−2.29e–5	0	−9.12e–4	−2.56e–4	−1.83e–3	−8.46e–4	−2.57e–3	−1.48e–3	−5.53e–4
4	4	4.95e–7	−1.30e–8	−2.00e–8	−1.04e–6	1.32e–7	−1.02e–8	−6.17e–5	−7.30e–5	−2.84e–5
5	5	1.07e–8	1.07e–9	2.24e–9	2.21e–8	3.03e–9	1.55e–9	−3.49e–5	2.08e–9	3.45e–6

**Table a2.** The *z*^′^ s for Song [9] UncExp.

***n***	***N***	***z*_3_^′^**	***z*_4_^′^**	***z*_51_^′^**	***z*_52_^′^**	***z*_61_^′^**	***z*_62_^′^**	***z*_71_^′^**	***z*_72_^′^**	***z*_73_^′^**
3	3	−2.29e–5	0	−1.52e–4	−1.28e–4	−7.63e–5	−1.41e–4	−2.15e–5	−6.18e–5	−4.61e–5
4	4	4.95e–7	−6.51e–9	−3.34e–9	−5.21e–7	5.49e–9	−1.70e–9	−5.14e–7	−3.04e–6	−2.37e–6
5	5	1.07e–8	5.33e–10	3.73e–10	1.10e–8	1.26e–10	2.58e–10	−2.91e–7	8.67e–11	2.87e–7

Comparing the *z*_3_^′^,*z*_4_^′^, *z*_51_^′^ and *z*_52_^′^ in Table a2 with those in Equations (65)–(67) of Song [9], we can find that the former is consistent with the later except for *z*_4_^′^ and *z*_51_^′^. (The *z*_4_^′^ and *z*_51_^′^ in Equations (66) and (67) of Song [9] are zero, while the *z*_4_^′^ and *z*_51_^′^ in Table a2 for UncExp4 and UncExp5 are near zero. The difference between *z*_4_^′^ and *z*_51_^′^ of Table a2 and those of Song [9] is due to round-off (to six places) in the Song [9] ς s used in [1].) This has been confirmed independently by a Reviewer of [1] that found identical results when using Song [9] equations and Song rounded ς s.

The authors wish to express their appreciation to a reviewer of [1] for his insightful comments and constructive suggestions used in the original article, also for his valuable suggestions used in this correction.
